# Overexpression of peptide deformylase in breast, colon, and lung cancers

**DOI:** 10.1186/1471-2407-13-321

**Published:** 2013-07-01

**Authors:** Harsharan Randhawa, Shireen Chikara, Drew Gehring, Tuba Yildirim, Jyotsana Menon, Katie M Reindl

**Affiliations:** 1 Department of Biological Sciences, North Dakota State University, Fargo, ND, USA; 2 Department of Biology, Faculty of Art and Science, Amasya University, Amasya, Turkey; 3 Ben May Department for Cancer Research, University of Chicago, Chicago, IL, USA

## Abstract

**Background:**

Human mitochondrial peptide deformylase (PDF) has been proposed as a novel cancer therapeutic target. However, very little is known about its expression and regulation in human tissues. The purpose of this study was to characterize the expression pattern of PDF in cancerous tissues and to identify mechanisms that regulate its expression.

**Methods:**

The mRNA expression levels of PDF and methionine aminopeptidase 1D (MAP1D), an enzyme involved in a related pathway with PDF, were determined using tissue panels containing cDNA from patients with various types of cancer (breast, colon, kidney, liver, lung, ovarian, prostate, or thyroid) and human cell lines. Protein levels of PDF were also determined in 2 colon cancer patients via western blotting. Colon cancer cells were treated with inhibitors of ERK, Akt, and mTOR signaling pathways and the resulting effects on PDF and MAP1D mRNA levels were determined by qPCR for colon and lung cancer cell lines. Finally, the effects of a PDF inhibitor, actinonin, on the proliferation of breast, colon, and prostate cell lines were determined using the CyQUANT assay.

**Results:**

PDF and MAP1D mRNA levels were elevated in cancer cell lines compared to non-cancer lines. PDF mRNA levels were significantly increased in breast, colon, and lung cancer samples while MAP1D mRNA levels were increased in just colon cancers. The expression of PDF and MAP1D varied with stage in these cancers. Further, PDF protein expression was elevated in colon cancer tissue samples. Inhibition of the MEK/ERK, but not PI3K or mTOR, pathway reduced the expression of PDF and MAP1D in both colon and lung cancer cell lines. Further, inhibition of PDF with actinonin resulted in greater reduction of breast, colon, and prostate cancer cell proliferation than non-cancer cell lines.

**Conclusions:**

This is the first report showing that PDF is over-expressed in breast, colon, and lung cancers, and the first evidence that the MEK/ERK pathway plays a role in regulating the expression of PDF and MAP1D. The over-expression of PDF in several cancers and the inhibition of cancer cell growth by a PDF inhibitor suggest this enzyme may act as an oncogene to promote cancer cell proliferation.

## Background

In prokaryotic organisms, the N-terminal methionine excision (NME) pathway is indispensible for proper protein functioning. This pathway involves two enzymes; peptide deformylase (PDF) which removes the formyl group from the initial methionine in nascent peptides, and methionine aminopeptidase (MAP) which subsequently removes the initial methionine [1]. Until recently, PDF was thought to exist only in prokaryotic organisms and hence has been the target of antimicrobial agents [2-5]. However, the recent discovery of PDF and a MAP isoform in the mitochondria of eukaryotes raises questions regarding their role in human cells [6-8].

Studies show that human PDF (HsPDF) can cleave the formyl group from an initiator methionine, but with reduced kinetics compared to the prokaryotic versions of the enzyme [2,8,9]. However, many of the respiratory Complex I peptides generated from mtDNA, putative substrates for PDF and MAP1D, retain their formylated initiator methionine [10]. In contrast, a recent report suggests that inhibition of PDF with actinonin results in reduced aerobic respiratory capacity by influencing the expression of proteins derived from the mtDNA [11].

While there are conflicting views for their role in NME in humans, it is likely PDF and MAP1D have alternative functions. Indeed, RNA interference of MAP1D altered anchorage-dependent growth of colon cancer cells [12] and inhibition of PDF with actinonin and numerous analogs decreased proliferation of many cancer cells while having minimal effects on non-cancer cell lines [13]. Further, PDF inhibitors resulted in a reduced tumor volume in a mouse xenograft model using HL-60 [14]. These results have lead to recent studies focused on the design of inhibitors to target PDF in cancer [14-16].

Despite these advances, little is known about the expression and regulation of the NME enzymes in cancers. MAP1D is over-expressed in colon cancer [12], but no study has reported the expression of PDF in cancerous compared to normal tissues. Further, no study has described a mechanism that regulates human PDF or MAP1D expression. Therefore, the purpose of this study was to identify the expression profiles of PDF and MAP1D in human cancers compared to normal tissues and to identify a signaling pathway involved in regulating their expression. Given the role of human PDF and MAP1D in cancer cell growth and adhesion, we hypothesized that these proteins would be up-regulated in cancer cells and tissues compared to normal and their expression would be modulated by growth-regulatory pathways. In this paper, we report that PDF is elevated in breast, colon, and lung cancer tissues and MAP1D is elevated in colon cancer tissue samples compared to non-cancer controls. We also show that PDF and MAP1D mRNA expression is down-regulated when MEK/ERK signaling is disrupted.

## Methods

### Cell culture

All cell lines, unless otherwise noted, were obtained from ATCC (Manassas, VA) and cultured at 37°C with 5% carbon dioxide. Hs578Bst normal breast cells were maintained in Hybri-Care Medium (ATCC) supplemented with 1.5 g/L sodium bicarbonate (Sigma; St. Louis, MO), 30 ng/ml mouse EGF (BD Biosciences; San Jose, CA), and 10% fetal bovine serum (FBS; Atlanta Biologicals; Lawrenceville, GA). Hs578T breast cancer cells were cultured in Dulbecco's Modified Eagle's Medium (DMEM; Thermo Scientific; Waltham, MA) supplemented with 0.01 mg/ml bovine insulin (Sigma) and 10% FBS. CCD-18Co normal colon cells were maintained in Eagle's Minimum Essential Medium (EMEM; ATCC) supplemented with 10% FBS. HT-29 colon cancer cells were cultured in McCoy’s 5a (Thermo Scientific) medium supplemented with 10% FBS. Hs888Lu normal lung fibroblasts and A549 lung cancer cells were cultured in DMEM plus 10% FBS. PrEC normal prostate epithelial cells were obtained from Cambrex Corporation (East Rutherford, NJ) and propagated in PrEGM media with Bulletkit growth supplements (Cambrex). PC-3 cells were grown in Ham’s F-12 K medium supplemented with 10% FBS.

### Human tissue samples and cDNA

TissueScan Cancer qPCR Arrays containing cDNA from normal and cancer tissue samples were purchased from Origene (Rockville, MA). The cDNA panels (cancer survey panel CSRT101, breast cancer panel BCRT101, matched colon cancer panel HCRT103, and matched lung cancer panel HLRT104), each had 48–96 samples per microplate. Equal loading of cDNA was verified by the manufacturer. Additionally, matched normal and colon cancer samples were obtained from two patients at the Veteran’s Affairs (VA) Hospital in Fargo, ND. This research was approved by the University of South Dakota and the North Dakota State University Institutional Review Board and performed according to the ethical guidelines imposed by these boards. Informed consent was obtained from each participant. Total RNA was isolated from human cell lines using the Fisher SurePrep Kit (Waltham, MA) and from human tissue samples using TRI Reagent (Molecular Research Center; Cincinnati, OH) as per the manufacturer’s suggestions. 100 ng of total RNA were reverse transcribed into cDNA using the qScript cDNA synthesis kit (Quanta Biosciences; Gaithersburg, MD).

### Signal transduction pathway inhibitors

HT-29 colon cancer cells were seeded into a 6 well plate at 1.5 million cells per well and incubated overnight. The next day, the cells were treated for 5 hours with 10 μM U0126, 10 μM LY294002, or 10 μM rapamycin (all from Cell Signaling). Total RNA or total protein was collected from the cells for further analysis.

### QPCR

Primers against human PDF and MAP1D were designed using Primer Express software (Applied Biosystems; Carlsbad, CA) and synthesized by Integrated DNA Technologies (Coralville, IA). Primer sequences were as follows; PDF forward AGGCGCTGTGTCGGGAGTGC, PDF reverse TCTCGCAGCCCTCGGGAAAG, MAP1D forward TATAGTTTTGCCGGCTGCAGT, MAP1D reverse ATGTGCTTAGGAACCGGATGA, β-actin forward CAGCCATGTACGTTGCTATCCAGG, β-actin reverse AGGTCCAGACGCAGGATGGCATG. Steady-state mRNA levels of PDF or MAP1D were determined for all cDNAs by real-time PCR using PerfeCTa SYBR Green FastMix (Quanta Biosciences). The cycling parameters were 95°C for 10 min followed by 40 cycles of 95°C for 30 sec and 60°C for 1 min and a dissociation program that included 95°C for 1 min, 55°C for 30 sec, and 95°C for 30 sec ramping up at 0.2°C/sec. One distinct peak was observed for the primer sets. For the cell lines, qPCR standards were prepared using human PDF and MAP1D full-length cDNA clones from Open Biosystems (Catalog numbers IHS1380-97652083 and MHS4426-99238965, Huntsville, AL). The 10^10^ molecules/μL standard was serially diluted to 10^2^ molecules/μL. The standards were run alongside the cDNA from the human cell lines in order to approximate the copy number of PDF or MAP1D in these cells. For the cDNA panels, fold-change in mRNA expression was calculated by comparing normalized threshold cycle numbers (C_T_) in the cancerous tissue compared to the normal tissues. The cell experiments were performed in triplicate.

### SDS-PAGE and western blotting

Cell pellets or human tissue samples from the VA Hospital were lysed using an SDS lysis buffer (Cell Signaling Technologies, Danvers, MA) containing protease and phosphatases inhibitors (Roche; Indianapolis, IN). Samples were briefly sonicated to dissociate cell membranes. Fifty μg of total protein isolated from the human cell lines or tissues were separated on 10% SDS-polyacrylamide gels at 100 V for 1 hr. Proteins were transferred to nitrocellulose membranes at 100 V for 75 min at 4°C. Blots were then probed overnight at 4°C with primary antibodies. The PDF antibody was a kind gift from Carmela Giglione and Thierry Meinnel (Centre National de la Recherche Scientifique, Gif-sur-Yvette, France). The MAP1D antibody was obtained from R&D Systems (Minneapolis, MN). The total and phosphor-ERK antibodies were purchased from Cell Signaling. The next day, blots were rinsed with 1X TBS-tween (0.1%) and probed with anti-rabbit secondary antibody (Jackson Immuno Research; West Grove, PA) for 1 hr at room temperature. The western blots were analyzed using SuperSignal West Pico Chemiluminescent Substrate (Thermo Fisher Scientific; Rockford, IL) and images captured using the MultiImage™ Light Cabinet (Alpha Innotech; San Leandro, CA). PDF levels were normalized to β-actin (Cell Signaling) expression. Immunoblots were performed in triplicate.

### Toxicity assay

Hs578Bst, Hs578T, CCD-18Co, HT-29, PrEC, and PC-3 cells were plated in 96-well microplates in growth medium at a density of 5,000 cells/well and incubated for 24 hours. The cells were then treated for 4 days with 0–250 μM actinonin. The CyQUANT (Life Technologies; Grand Island, NY) cell proliferation assay was performed according to the manufacturer’s instructions. Fluorescent readings were taken on day 4 to determine the percentage of viable cells. Each condition was performed with eight replicates, and the experiments were repeated three times.

### Statistical analysis

SigmaPlot v12 software (Systat Software Inc.; Chicago, IL) was used for all statistical analyses. For all tests, a p-value cut-off of < 0.05 was used to determine statistical significance. For the cell lines, the PDF and MAP1D values were related to the standard curves for the respective targets to yield the approximate mRNA copy number/cell. These values were then normalized to β-actin values. The data are expressed as the average copy number ± SD for 3 replicates. A t-test comparing the PDF or MAP1D mRNA copy number in the cancer cell lines to the copy number in their respective normal cell lines. For the cancer tissue cDNA plates, the average Ct value for all of the non-cancer tissue samples was set to 1. The data are expressed as the relative fold change in each individual sample compared to the average of these controls. A t-test was run for PDF mRNA expression in the cancer survey samples compared to their non-cancer controls. One-way ANOVA on ranks was done using Dunn’s method for multiple comparisons in the cancer stage I-III breast, colon, and lung samples compared to their normal tissues. A paired t-test was done to compare the effect of actinonin on the proliferation of the cancer cell line to the normal cell line. The data represent the percentage of viable cells ± SD for 8 replicates. Finally, a t-test was used to determine the effect of U0126 on the expression of PDF and MAP1D mRNA in 3 independent replicates.

## Results

### PDF and MAP1D expression is elevated in human cancer cell lines

**Figure 1:**
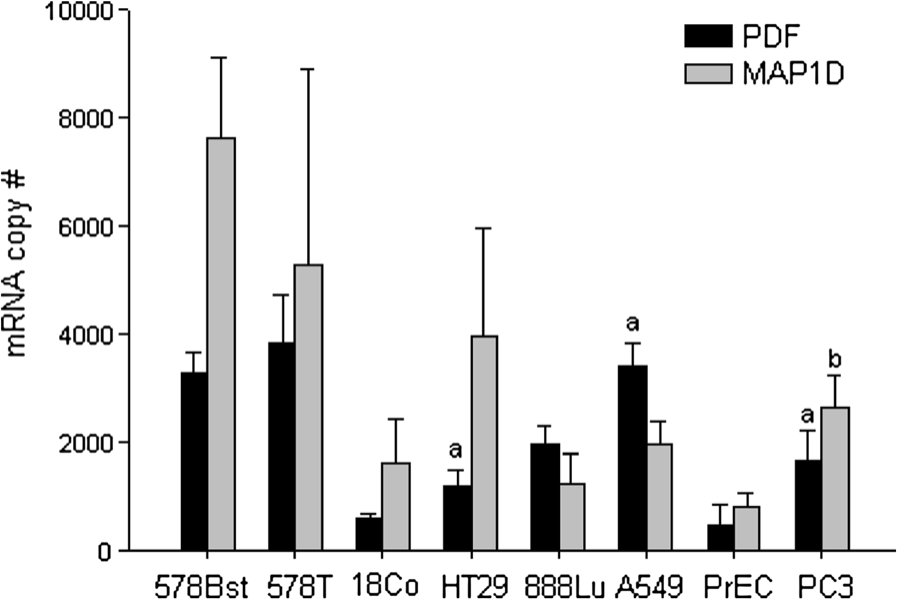
**PDF and MAP1D mRNA expression varies across human cell lines.** The expression of PDF and MAP1D mRNA in normal breast (Hs578Bst), colon (CCD-18Co), lung (Hs888Lu), and prostate (PrEC) cell lines was compared to the expression in breast cancer (Hs578T), colon cancer (HT29), lung cancer (A549), and prostate cancer (PC3) cell lines, respectively. The mRNA copy numbers for PDF and MAP1D were related to cDNA standards for these two genes and then normalized to β-actin levels. PDF was significantly (denoted by “a”) elevated in the colon, lung, and prostate cancer cell lines compared to their respective normal cell lines while MAP1D was significantly (denoted by “b”) elevated only in the prostate cancer cell line. The data represent the average mRNA copy number for 3 replicate experiments ± SD.

We compared the expression of PDF and MAP1D in four different types (breast, colon, lung, and prostate) of cancer cell lines to non-cancer cell lines. PDF mRNA expression was significantly higher in the HT-29 colon, A549 lung, and PC-3 prostate cancer cell lines compared to the CCD-18Co colon, Hs888Lu lung, and PrEC prostate non-cancer cell lines (Figure 1). MAP1D was significantly elevated in the PC-3 compared to PrEC cell line, but was not significantly different in the other pairs of cell lines (Figure 1). The Hs578Bst and Hs578T cell lines are a normal breast and breast cancer cell line isolated from the same patient. These cell lines did not significantly differ in their PDF or MAP1D expression, although PDF was slightly elevated and MAP1D was reduced. The data suggest that PDF and MAP1D expression varies across cell type and that they show altered expression in cancer compared to non-cancer cells.

### Actinonin inhibits the growth of both cancer and non-cancer cell lines

**Figure 2:**
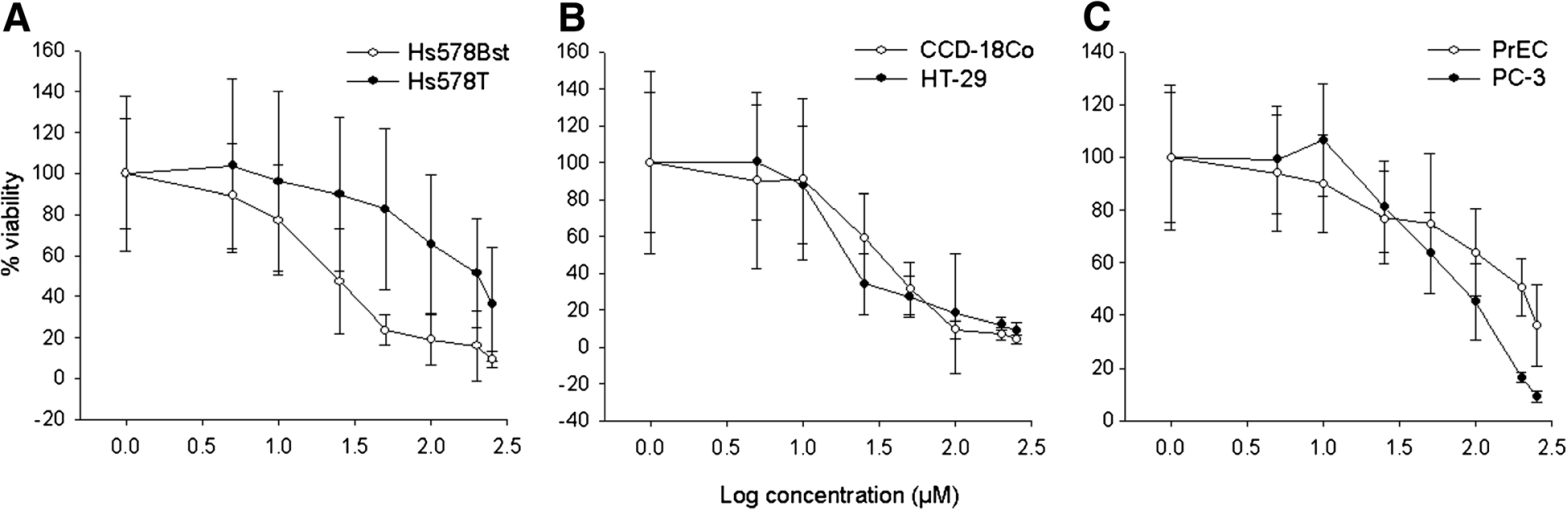
**Actinonin inhibits the growth of cancer cell lines to a greater degree than non**-**cancer cell lines. (A)** Normal breast (Hs578Bst) and breast cancer (Hs578T), **(B)** normal colon (CCD18Co) and colon cancer (HT29), and **(C)** normal prostate (PrEC) and prostate cancer (PC3) cell lines were treated with 0–250 μM actinonin for 96 hrs before the percentage of viable cells was determined. The growth-inhibitory effect of actinonin was significantly greater in the breast cancer and prostate cancer cell lines than in their non-cancer control cell lines. The data represent the percentage of viable cells ± SD for 3 experiments with 8 replicates each.

The effect of the PDF inhibitor actinonin on the proliferation capacity of colon, breast, and prostate cancer and non-cancer cell lines was measured. Actinonin inhibited the proliferation of both cancer and non-cancer cell lines in a concentration-dependent manner, but had greater inhibition of cell proliferation in cancer cells compared to non-cancer cells (Figure 2A-C). The IC_50_’s were 19.3, 17.3, and 113.5 μM for the Hs578T, HT-29, and PC-3 cancer cell lines, respectively while the IC_50_’s were 208, 31.9, and 207.4 μM for the Hs578Bst, CCD-18Co, and PrEC cells, respectively. While the IC_50_ was higher in the normal colon compared to the colon cancer cell line, the difference in the percentage of viable cells was not statistically significant. In contrast, actinonin significantly affected the growth of breast and prostate cancer cells compared to their non-cancer cell controls. In general, the data suggest that inhibition of PDF by actinonin has a greater effect on proliferation of cancer cells compared to normal cells.

### PDF mRNA is elevated in many cancer tissues

**Figure 3:**
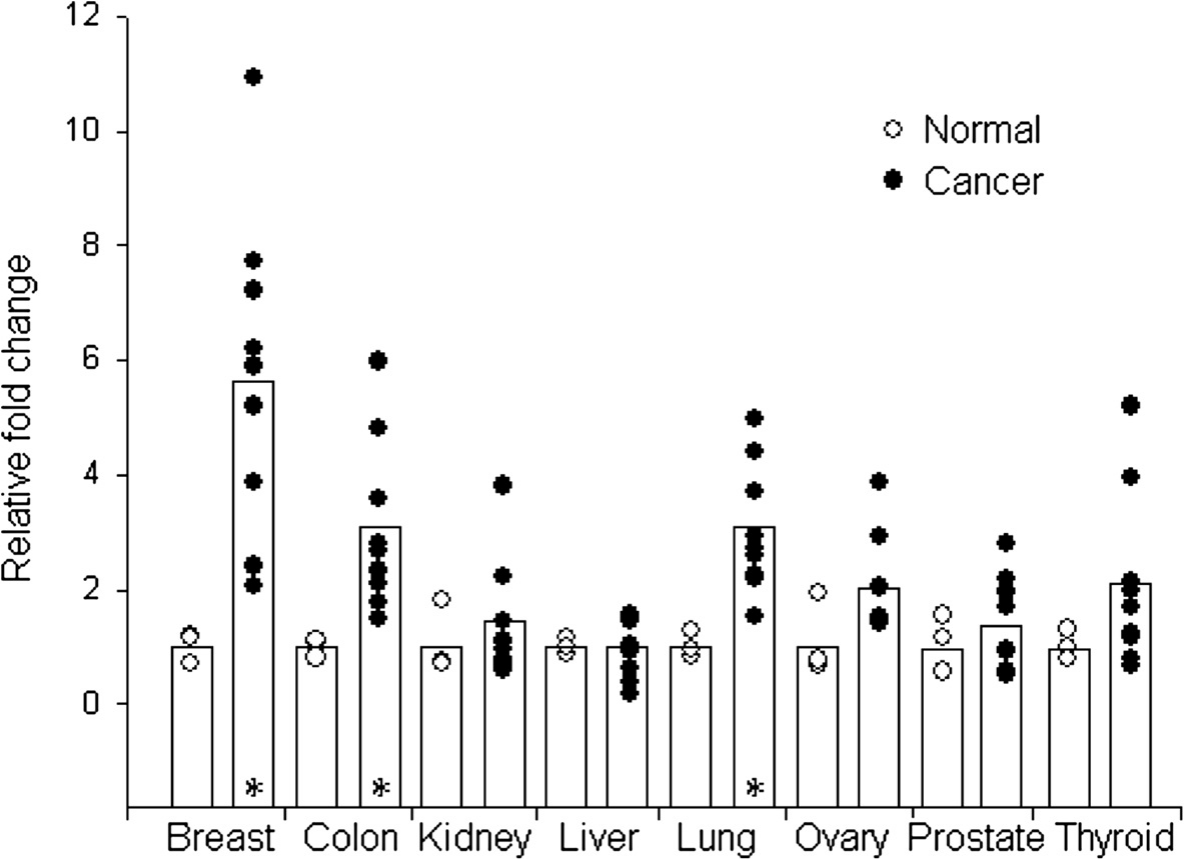
**PDF mRNA is significantly elevated in breast, colon, and lung cancer.** With the exception of liver, that showed equal expression, PDF was elevated in all cancers (●) compared to non-cancer/normal (○) tissues. All stages of disease were pooled for the cancer groups. Statistically significant differences (*) were observed in breast, colon, and lung cancers with expression values 5.8, 3.5, and 3.4-fold higher in cancer compared to normal tissues, respectively.

TissueScan^TM^ Cancer qPCR Arrays containing cDNA from 96 tissue samples representing eight different cancers (breast, colon, kidney, liver, lung, ovary, prostate, thyroid) were used to determine PDF expression in cancer compared to non-cancer tissues. For each tissue type, the array contained 3 normal control tissues and 9 cancer tissues. With the exception of liver cancer that showed no change compared to control liver samples, PDF was at least slightly elevated in all cancer tissues compared to control, and PDF mRNA levels were significantly elevated in the breast, colon, and lung cancer tissue samples compared to their non-cancer samples (Figure 3). Breast cancer showed a 5.8-fold increase in expression of PDF while colon and lung showed a 3.5 and 3.4-fold increase in PDF expression, respectively.

**Figure 4:**
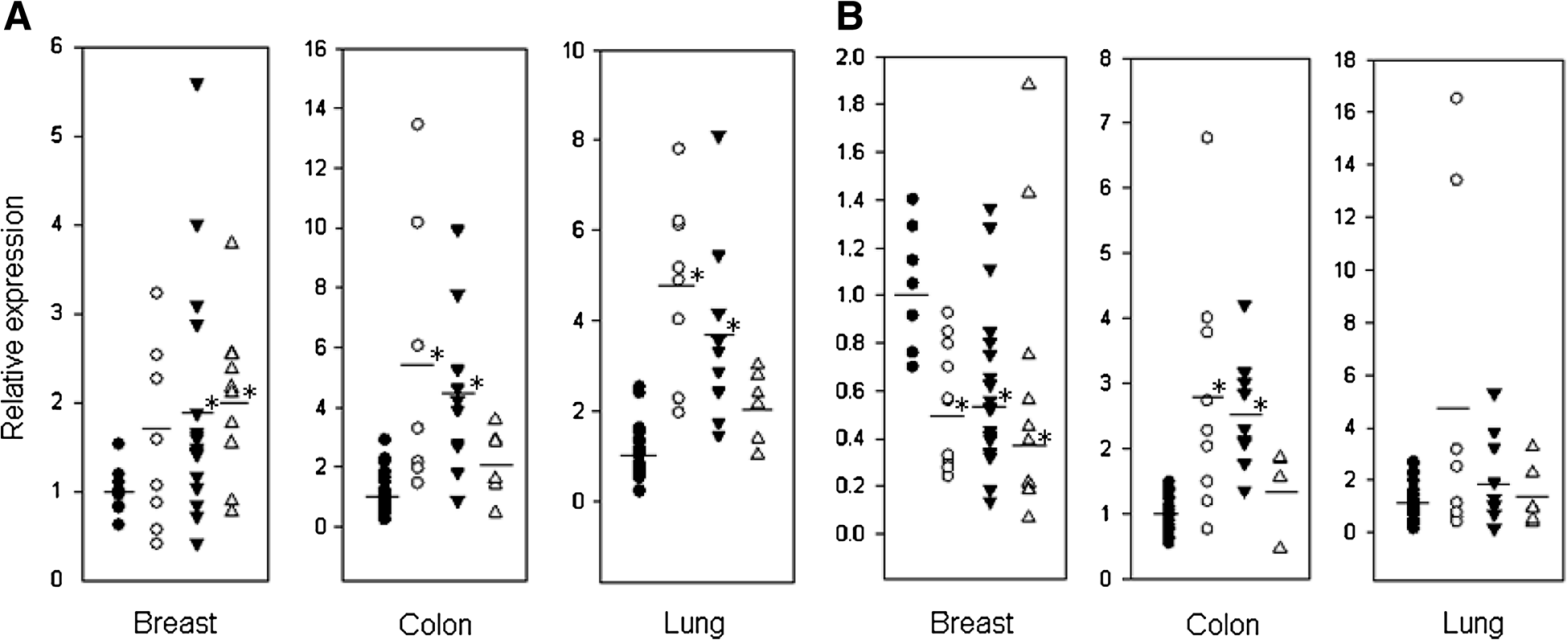
**PDF and MAP1D mRNA expression varies with stage in breast, colon, and lung cancer samples. (A)** PDF and **(B)** MAP1D mRNA expression is shown for normal (●) tissues relative to stage I (○), stage II (▼), and stage III (Δ) tissues for breast, colon, and lung cancer patients. PDF levels are significantly (*) elevated in late-stage breast, and early-stage colon and lung cancers while MAP1D levels are significantly increased in early-stage colon cancer, but decreased in breast cancer.

Additional tissue panels for breast, colon, and lung cancer patients were used to validate the previous results and to assess MAP1D levels in these cancer types. Colon and lung tissue panels contained 48 matched normal and cancer tissue samples from 24 cancer patients while the breast tissue panels contained 48 unmatched tissue samples that included 12 normal breast tissue controls and 36 breast cancer samples at various disease stages. Similar to the first results, PDF was elevated in breast, colon, and lung cancer samples and showed stage-dependent expression with the highest expression in late stage breast cancer, but early stage colon and lung cancers (Figure 4A). MAP1D mRNA expression was elevated in early-stage colon cancer samples, and was surprisingly reduced in breast cancer samples compared to control samples (Figure 4B). There was no significant change in MAP1D mRNA levels in lung cancer samples at any stage compared to control. These results suggest PDF and MAP1D expression is altered in certain cancer tissues and that expression of these enzymes is correlated with the stage of disease.

### PDF protein levels are elevated in colon cancer tissues

**Figure 5:**
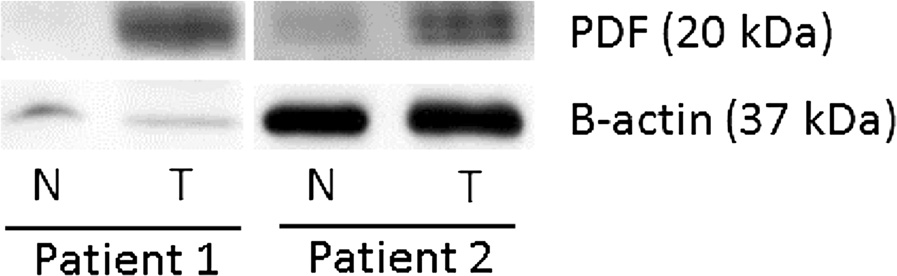
**PDF protein expression is elevated in colon tumor tissues.** Western blotting was done to determine the expression of PDF in colon cancer tissue samples (T) relative to normal colon tissue (N) from two patients. Elevated PDF levels were found in the colon tumor samples for each patient. A β-actin antibody was used to confirm equal protein loading of the tissue samples for each patient. Two replicate experiments were performed and this image shows one representative experiment.

To verify that the increased PDF mRNA levels translated to increased PDF protein levels, we screened two sets of colon cancer tissues for PDF expression. Matched colon cancer and normal colon tissue sets were obtained from two patients at the VA Hospital in Fargo, ND in accordance with IRB policies. Western blotting for PDF revealed a striking elevation of PDF expression in the tumor sample of both of these patients relative to their matched normal colon tissue (Figure 5).

### Inhibition of MEK/ERK results in reduced expression of PDF and MAP1D in colon cancer cells

**Figure 6:**
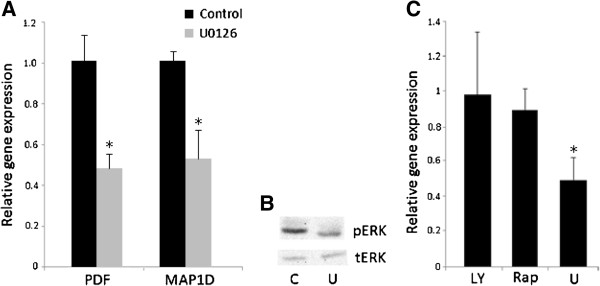
**Inhibition of MEK down-regulates PDF and MAP1D mRNA expression in colon cancer cells. (A)** Treatment of HT-29 colon cancer cells with 10 μM U for 5 hr resulted in about a 50% reduction in both PDF and MAP1D mRNA expression. **(B)** Western blot analysis confirmed that 5 hr treatment of HT-29 colon cancer cells with 10 μM U reduced phosphorylated ERK (pERK) levels. Total ERK (tERK) expression was determined in order to show equal protein loading. **(C)** Treatment of HT-29 cells with other inhibitors Ly294002 (LY) and rapamycin (Rap) does not affect PDF mRNA expression. These experiments were repeated 3 times and the data represent the average relative gene expression in the inhibitor-treated cells relative to the vehicle-treated controls. Statistically significant (p < 0.05) differences are denoted by *.

The regulation of PDF or MAP1D expression in human cells has not been previously studied. To understand potential mechanisms that regulate PDF and MAP1D gene expression, we used pharmacological inhibitors to target the MEK/ERK, PI3K, and mTOR signaling pathways and determined their effects on PDF or MAP1D expression. Treatment of HT-29 colon cancer cells with the MEK inhibitor U0126 resulted in a 51% reduction in expression of PDF mRNA and a 47% reduction in MAP1D (Figure 6A). Western blotting confirmed that U0126 inhibited ERK signalling these cells (Figure 6B). Unlike U0126, the PI3K inhibitor LY294002 and mTOR inhibitor rapamycin did not have an effect on PDF expression in HT-29 cells (Figure 6C).

## Discussion

PDF and MAP are essential enzymes in prokaryotic peptide synthesis, but their role in eukaryotic cells is less appreciated. Previous studies have suggested PDF and MAP1D as therapeutic targets for cancer treatment given their roles in modulating cell proliferation, adhesion, and aerobic respiration [11-13]. As a result, the goal of this research was to characterize the expression pattern of PDF and MAP1D in human cancer tissues in order to better understand their potential roles in these cancers.

Over-expression of MAP1D has been previously observed in colon cancer tissues; 7 out of 8 colon cancer patients showed increased MAP1D mRNA expression and 9 out of 12 patients showed increased MAP1D protein expression [12]. Similarly, we also found that MAP1D was elevated in colon cancers, but not lung cancers. Interestingly we found that MAP1D mRNA expression was significantly reduced in breast cancer samples compared to normal breast tissue. This is the first report to suggest PDF is over-expressed in cancer, particularly breast, colon, and lung. Stage-dependent expression of PDF was observed in the tissue samples where higher expression was found in early stages of colon and lung cancer, but later stages of breast cancer. Early expression of PDF indicates it plays a role in the proliferation of tumor cells. The over-expression of PDF and MAP1D, particularly in early-stage colon cancer, suggests that these enzymes are important for cancer cell growth.

PDF and MAP1D are encoded in the nuclear genome (chromosome 16 and 2, respectively) and translocate to mitochondria [14]. It was interesting to find that the expression of both HsPDF and MAP1D was regulated by a similar pathway. Use of the MEK inhibitor U0126 resulted in about a 50% reduction in PDF and MAP1D expression in a human colon cell line. Conversely, rapamycin and LY294002 had little effect on PDF expression suggesting the MEK/ERK pathway specifically contributes to the expression of NME enzymes. A genetic and functional linkage of PDF and MAP1D has been shown in other animal genomes suggesting the tight regulation of NME activity in eukaryotic mitochondria [8]. The involvement of a growth-regulatory pathway in modulating PDF expression, provides further support that PDF promotes the growth of tumors and lends support to the pursuit of PDF inhibitors as cancer therapies.

Lee *et al*. showed that the PDF inhibitor actinonin selectively inhibited the proliferation of numerous cancer cell lines while having a minimal effect on the growth of non-cancer cell lines [13]. Similarly, our data show that actinonin had significantly greater growth-inhibitory effects on breast and prostate cancer cells than non-cancer cell lines. These results suggest that PDF does play a role in the growth of cancer cells and may offer a selective target for cancer treatment.

## Conclusions

In conclusion, we found that PDF is up-regulated in several cancer types including breast, colon, and lung. Our data suggest that the MEK/ERK pathway contributes to the expression of PDF and MAP1D colon cancer cells. Finally, we demonstrated that the PDF inhibitor actinonin inhibits the growth of cancer cell lines to a greater degree than non-cancer cell lines. These data suggest that PDF and MAP1D may function as oncogenes to promote tumor development and are potential selective targets for colon cancer therapy.

## Abbreviations

(ERK): Extracellular-signal-regulated kinase; (MAP): Methionine aminopeptidase; (MEK): Mitogen-activated protein kinase kinase; (mtDNA): Mitochondrial DNA; (mTOR): Mammalian target of rapamycin; (NME): N-terminal methionine excision; (PDF): Peptide deformylase; (PI3K): Phosphatidylinositol 3-kinase.

## Competing interests

The authors have no competing interests in relation to this paper.

## Authors’ contributions

KR conceived, designed, and performed mRNA expression and actinonin experiments, analyzed the data, and wrote the manuscript. HR and SC performed mRNA and protein expression experiments, analyzed the data, and wrote the manuscript. DG and TY conducted gene regulation experiments using the signaling molecule inhibitors and analyzed the data. JM assisted with the design of the expression and regulation experiments and data analysis. All authors read and approved the final manuscript.

## Pre-publication history

The pre-publication history for this paper can be accessed here:

http://www.biomedcentral.com/1471-2407/13/321/prepub
